# Comparison of the Immune Responses to Different Formulations of BC02-Adjuvanted HPV Types 16 and 18 Bivalent Vaccines in Mice

**DOI:** 10.3390/vaccines11101553

**Published:** 2023-09-30

**Authors:** Junli Li, Huicong Xie, Lili Fu, Xiaonan Guo, Jiaxin Dong, Miao Xu, Guozhi Wang, Aihua Zhao

**Affiliations:** 1 Division of Tuberculosis Vaccine and Allergen Products, Institute of Biological Product Control, National Institutes for Food and Drug Control, Beijing 102629, China; lijunli@nifdc.org.cn (J.L.); congxie10241@163.com (H.X.); flili2007@126.com (L.F.); xnguojlu@163.com (X.G.); dongjx000@163.com (J.D.); xumiaobj@126.com (M.X.); tbtestlab@nifdc.org.cn (G.W.); 2 Key Laboratory for Quality Research and Evaluation of Biological Products, National Medical Products Administration (NMPA), Beijing 102629, China; 3 Key Laboratory of Research on Quality and Standardization of Biotech Products, National Health Commission (NHC), Beijing 102629, China

**Keywords:** HPV prophylactic vaccine, compound adjuvant, adjuvant formulation, vaccine formulation, neutralizing antibody

## Abstract

To achieve maximum efficacy, vaccines, such as subunit, recombinant, and conjugate vaccines, necessitate the incorporation of immunostimulators/adjuvants. Adjuvants play a vital role in bolstering and extending the strength of the immune response while also influencing its type. As antigen and adjuvant formulations become more intricate, it becomes imperative to establish a well-characterized and robust formulation to ensure consistent and reproducible outcomes in preclinical and clinical studies. In the present study, an HPV bivalent vaccine was developed using a BC02 adjuvant in conjunction with HPV 16 and 18 L1 VLP antigens produced from an E. coli expression system. The study involved evaluating the adjuvant formulation and in vivo immunogenicity in mice. Remarkably, a medium-dose of BCG-CpG-DNA combined with a low-dose of aluminum hydroxide substantially enhanced the immunogenicity of HPV16 and 18 VLPs, resulting in improved cellular and humoral immune responses.

## 1. Introduction

Human papillomaviruses (HPV) belong to the papillomaviridae family and are double-stranded DNA viruses that infect humans. There are over 100 identified types of HPV, categorized into cutaneous or mucosal types based on their tissue tropism [1]. They are further classified as low-risk (LR) or high-risk (HR) according to their oncogenic potential [2]. HPV infection is related to various cancers, including cervical cancer, and is an etiological factor in anus (90–93%), oropharynx (12–63%), penis (36–40%), vagina (40–64%), and vulva (40–51%) cancers [3]. Although HPV infection is usually asymptomatic, individuals with compromised immune systems, older age, or multiple sexual partners may experience latent viral reactivation, leading to the progression of viral lesions to invasive cancer. Approximately 63–95% of noncervical cancers and 70–76% of cervical cancers are caused by HR HPV types 16 and 18, underscoring the importance of prophylactic HPV vaccination.

The first HPV vaccine was licensed in 2006, and several bivalent, quadrivalent, and nonavalent vaccines have been approved since 2022 [4]. Among them, Gardasil™, Cervarix™, and Gardasil-9™ are widely licensed in most countries. Gardasil™ (Merck, approved in 2006) is a quadrivalent vaccine comprising virus-like particles (VLPs) of LR viruses (HPV types 6 and 11) as well as HR viruses (HPV types 16 and 18), which account for 90% of genital warts [5]. Cervarix™ (GlaxoSmithKline, approved in 2009) is a bivalent vaccine with VLPs of HR viruses (HPV types 16 and 18), which account for 70% of invasive cervical cancer cases globally [6]. Gardasil-9™ (Merck, approved in 2014) is a second-generation vaccine comprising VLPs of two LR viruses (HPV types 6 and 11) as well as seven HR viruses (HPV types 16, 18, 31, 33, 45, 52, and 58) [7,8]. Currently, the FDA has extended its approval to include eligible females and males aged between 27 and 45 years for Gardasil-9™, aiming to prevent HPV-associated cancers and disorders. All three anti-HPV vaccines contain HR HPV types 16 and 18. They are manufactured using baculovirus-infected insect cell Trichoplusia for Cervarix™, while Saccharomyces cerevisiae expresses the L1 gene for Gardasil™ and Gardasil-9™. There are additional distinctions between the vaccine types. Cervarix™ includes the AS04 adjuvant (50 μg 3-O-desacyl-4′ monophosphoryl lipid A (MPL) and 500 μg aluminum hydroxide), while Gardasil™ and Gardasil-9™ contain either 225 mg or 500 mg amorphous aluminum hydroxyphosphate sulfate (AAHS). Both AS04 and AAHS-compatible vaccines have demonstrated good immunogenicity and safety profiles. Nevertheless, bivalent vaccines have been shown to be more effective against HPV infection, providing 6.4 years of protection compared to quadrivalent vaccines, which offer 5 years of protection [9]. The different adjuvant systems might be partly responsible for this observed difference in efficacy. The prophylactic vaccines have shown close to 100% protection against lesions caused by an incident HPV infection [10,11,12]. However, high costs limit accessibility in developing countries, where cervical cancer remains a significant health concern [13,14]. In 2017, it was reported that 11.9% of global cervical cancer deaths took place in China [15]. Implementing regional vaccine production could lead to a cost reduction and help meet the demand for vaccines more effectively.

The BC02, also known as the BCG-CpG-DNA Compound Adjuvant System 02, is a compound adjuvant composed of an inorganic salt adjuvant, aluminum hydroxide (Al(OH)_3_), combined with a fragment of DNA from the Bacillus Calmette-Guerin genome that contains an unmethylated CpG motif. In the present study, we assessed the humoral and cellular immune responses in mice to *E. coli*-derived HPV16 and HPV18 L1 VLPs using different formulations of the BC02 compound adjuvant. The primary goal was to determine the most optimal compatibility dose of BC02 adjuvant with HPV16 and 18 VLPs and to evaluate the proprietary BC02 as a potential candidate adjuvant for the bivalent HPV vaccine.

## 2. Materials and Methods

### 2.1. Animal Ethical Statement

All animal-related procedures were conducted at the Institute for Laboratory Animal Resources of the National Institute of Food and Drug Control (NIFDC) under strict adherence to ethical considerations. The Institutional Animal Care and Use Committee of NIFDC granted approval for all laboratory animal experiments. The health of the mice used in the study was maintained under a specific-pathogen-free (SPF) condition, and they were housed in a constant environment with regulated temperature, humidity, and a 12 h light/dark cycle. Before immunization, the mice were given three days to acclimate to their surroundings. Throughout the study, the mice were provided with unrestricted access to food and water and received attentive care from specialized personnel.

### 2.2. Biological Components and Reagents

In our laboratory, we developed and preserved the following biological components and reagents: BCG-CpG-DNA novel biological adjuvant, BCG-CpG-DNA compound adjuvants system 02 (BC02), HPV 16/18 L1 virus-like particles (VLPs), and human embryonic kidney cells (HEK 293; Invitrogen, Carlsbad, CA, USA). Additionally, we obtained aluminum hydroxide (Alhydrogel^®^) from InvivoGen (Grand Island, NE, USA) and procured the following materials from Gibco (Grand Island, NE, USA): phosphate-buffered saline (PBS), Dulbecco’s modified Eagle’s medium (DMEM), RPMI-1640 medium, 100 × Penicillin-Streptomycin, and fetal bovine serum (FBS). Furthermore, IL-2 and IFN-γ ELISpot kits were supplied by Mabtech (Nacka Strand, Sweden).

### 2.3. Immunization and Sample Collection

**Figure 1:**
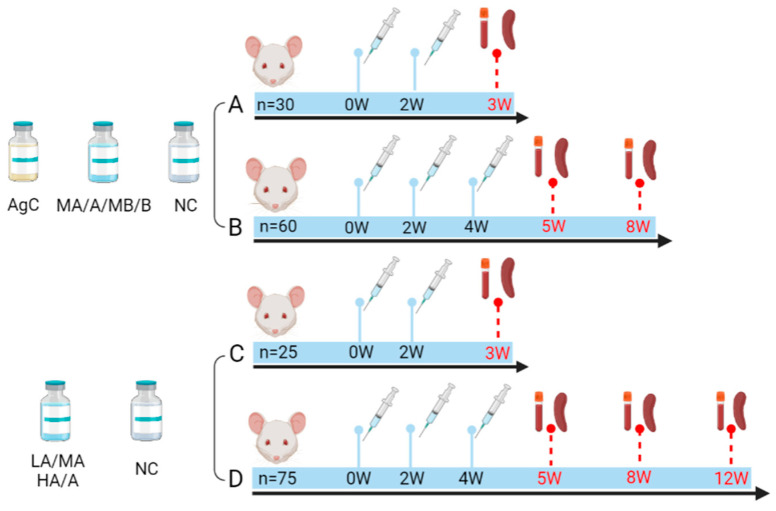
Immunization and sample collection timeline. At the designated time points, mice were euthanized, and spleen and peripheral blood samples were collected. Subsequently, sera and spleen lymphocytes were isolated for immunological analyses. n: number of immunized mice, W: week.

Female BALB/c mice, aged six to eight weeks and weighing 20–22 g, were included in the study. The mice were housed in SPF conditions. They were randomly allocated into different groups, each containing 5 mice (Table 1). Intramuscular-injection (i.m.) immunization was given either twice or three times at a 2 week interval, based on the group assignment and the specific vaccine schedule detailed in Figure 1. Negative control (NC) groups received injections of the same dose of 1× PBS. At the designated time points, the mice were euthanized, and their spleen and peripheral blood were collected. Peripheral blood specimens were allowed to clot at 4 °C for 8 h prior to centrifugation at 1200× *g* for 10 min. The resulting sera were assigned to smaller portions and stored at −80 °C. The spleen was aseptically dissected, and splenic lymphocytes were isolated through density gradient centrifugation following gentle trituration. Immunological analysis was carried out promptly on the isolated lymphocytes.

### 2.4. IL-2 and IFN-γ ELISpot Assays

The abundance of mouse splenocytes producing IL-2 and IFN-γ was measured using enzyme-linked immunospot (ELISpot) assays, following the kit’s instructions. Briefly, a single-cell suspension was prepared from the spleens of immunized mice at a concentration of 5.0 × 10^6^ cells/mL. Subsequently, 50 µL of the cell suspension was grown in duplicate on an ELISpot plate with antibody coating. The cells were then exposed to HPV types 16 or 18 L1 VLPs at a dose of 3 µg each, culture medium (negative control), or concanavalin A (0.1 µg, positive control) for 48 h at 37 °C in a 5% CO_2_ atmosphere. The ELISpot kit was used to detect IFN-γ and IL-2-secreting cells, and positive spots were determined using a UV microanalyzer (CTL-ImmunoSpot^®^ S5; Cellular Technology, Kennesaw, GA, USA). 

### 2.5. Preparation of HPV Pseudovirions

HEK 293 cells were evenly distributed into a T-175 flask, followed by incubation for 16 h. Subsequently, they were transfected using Lipofectamine 2000 reagent (Invitrogen, USA). The L1 gene-containing plasmids were co-transfected into HEK 293 cells together with a reporter plasmid that encodes secreted alkaline phosphatase (pYSEAP). Following a 72 h incubation period, the cells were lysed and incubated overnight at 37 °C. The unrefined pseudovirions were dissolved by adding 5 M NaCl and then centrifuged at 1500× *g* for 10 min at 4 °C. The purified pseudovirions were examined through 12% SDS-PAGE gels and titrated on HEK 293 cells to assess infectivity using SEAP analysis. The purified pseudovirions were subsequently pooled and stored at −80 °C until further use.

### 2.6. Pseudovirus-Based Neutralization Assays

HEK 293 cells (1.5 × 10^5^ cells/well) were pre-plated in a 96-well plate with 100 µL DMEM containing 1% penicillin-streptomycin and 10% FBS for 4–6 h. Purified HPV 16 or HPV 18 pseudovirions were subjected to a 2000-fold dilution. The diluted pseudovirion stock (60 µL/well) was added with 60 µL series diluted serum (1:20 to 1:327,680) in a 96-well non-treated plate and then incubated at 4 °C for 1 h. Subsequently, 100 µL of the pseudovirion-serum mixture was transferred onto the pre-plated cells, followed by incubation for 72 h at 37 °C under a 5% CO_2_ atmosphere. After that, 100 µL of supernatant was collected and centrifuged at 500× *g* for 5 min. The SEAP content in 15 µL of the supernatant was measured using the Great ESCAPE SEAP Chemiluminescence Kit (Clontech, CA, USA). After adding the substrate for 20 min, each sample was analyzed using a 1420 multilabel counter (VICTOR3). Neutralization activity was determined by identifying the highest serum dilution that led to a >50% reduction in SEAP activity.

### 2.7. Statistical Methods

Statistical tests were conducted with GraphPad Prism v8.0. To assess differences among multiple groups, one-way ANOVA and Tukey’s multiple comparison test were employed. All values are presented as the mean ± S.E. Statistical significance was set at *p* < 0.05.

## 3. Results

### 3.1. Addition of BCG-CpG-DNA Reduced Required Al(OH)_3_ Dosage in BC02 Compound Adjuvant

In this study, mice were administered two doses of vaccine containing 2 μg HPV16 and 18 L1 VLPs with or without the adjuvants low-dose (25 µg) aluminum hydroxide (formulation A) or high-dose (50 µg) aluminum hydroxide (formulation B) compatible with BCG-CpG-DNA at a 2 week interval.

**Figure 2:**
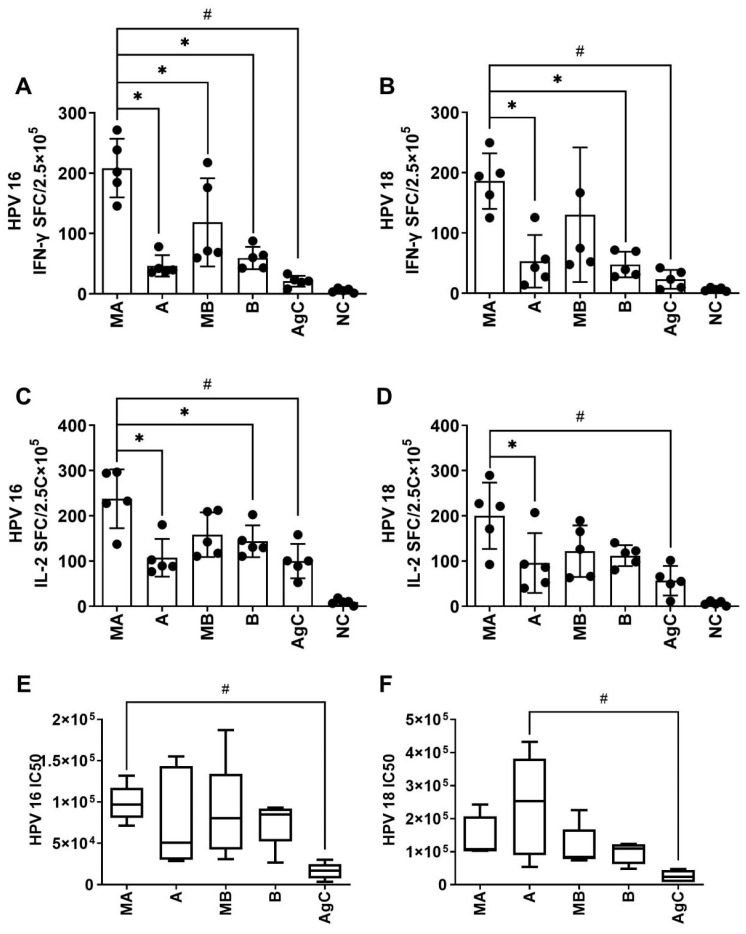
Two doses of different adjuvant combination vaccines induced an immune response to HPV types 16 and 18 L1 VLP antigens. The mice were immunized two times intramuscularly (i.m.) during a period of 2 weeks, either with HPV types 16 and 18 L1 VLPs alone or in combination with adjuvants. PBS was administered to mice in the negative control group. Spleen lymphocytes and sera were collected 1 week following the second immunization. (**A**,**B**): Both HPV16 and HPV18 L1 VLP-specific IFN-γ-producing splenocytes were evaluated using ELISpot assays. (**C**,**D**): Both HPV16 and HPV18 L1 VLP-specific IL-2-producing splenocytes were assessed by ELISpot assays. (**E**,**F**): Sera HPV16 and 18 antibody titers were detected using pseudovirus-based neutralization assays. * and # indicate a significant difference between adjuvanted vaccines or between adjuvanted vaccines and unadjuvanted antigens, respectively. *p* < 0.05.

The results showed that all mice receiving the adjuvants exhibited an obvious increase in HPV16-specific IFN-γ and IL-2 secretion (Figure 2A,C). Notably, the MA group exhibited the highest values compared to other groups (MA vs. A _(16-IFN-γ)_: *p* < 0.0001 and MA vs. A _(16-IL-2)_: *p* = 0.0009; MA vs. B _(16-IFN-γ)_: *p* < 0.0001 and MA vs. B _(16-IL-2)_: *p* = 0.0227; MA vs. AgC _(16-IFN-γ)_: *p* < 0.0001 and MA vs. AgC _(16-IL-2)_: *p* = 0.0005). Similarly, HPV18-specific IFN-γ and IL-2 secretion were also remarkably increased in all groups receiving the adjuvants (Figure 2B,D). The MA group showed the highest response compared to other groups (MA vs. A _(18-IFN-γ)_: *p* = 0.0073 and MA vs. A _(18-IL-2)_: *p* = 0.0286; MA vs. B _(18-IFN-γ)_: *p* = 0.0050; MA vs. AgC _(18-IFN-γ)_: *p* = 0.0008; and MA vs. AgC _(18-IL-2)_: *p* = 0.0014). Low-dose aluminum hydroxide and BCG-CpG-DNA appear to promote a Th1-type immune response to HPV types 16 and 18 L1 VLPs.

Moreover, the addition of BC02 to HPV16 L1 VLP antigens stimulated the humoral response, leading to a 7.3-fold increase in antibody titer compared to antigen alone (MA vs. AgC _(16)_, Figure 2E). The level of anti-HPV18 L1 VLP antibodies was increased 9.8-fold by the addition of alum (A vs. AgC _(18)_, Figure 2F), but the differences were not significant compared with BC02-adjuvanted groups.

### 3.2. Immune Response to HPV16 and 18 L1 VLPs after Three Doses of BC02-Adjuvanted Vaccines

**Figure 3:**
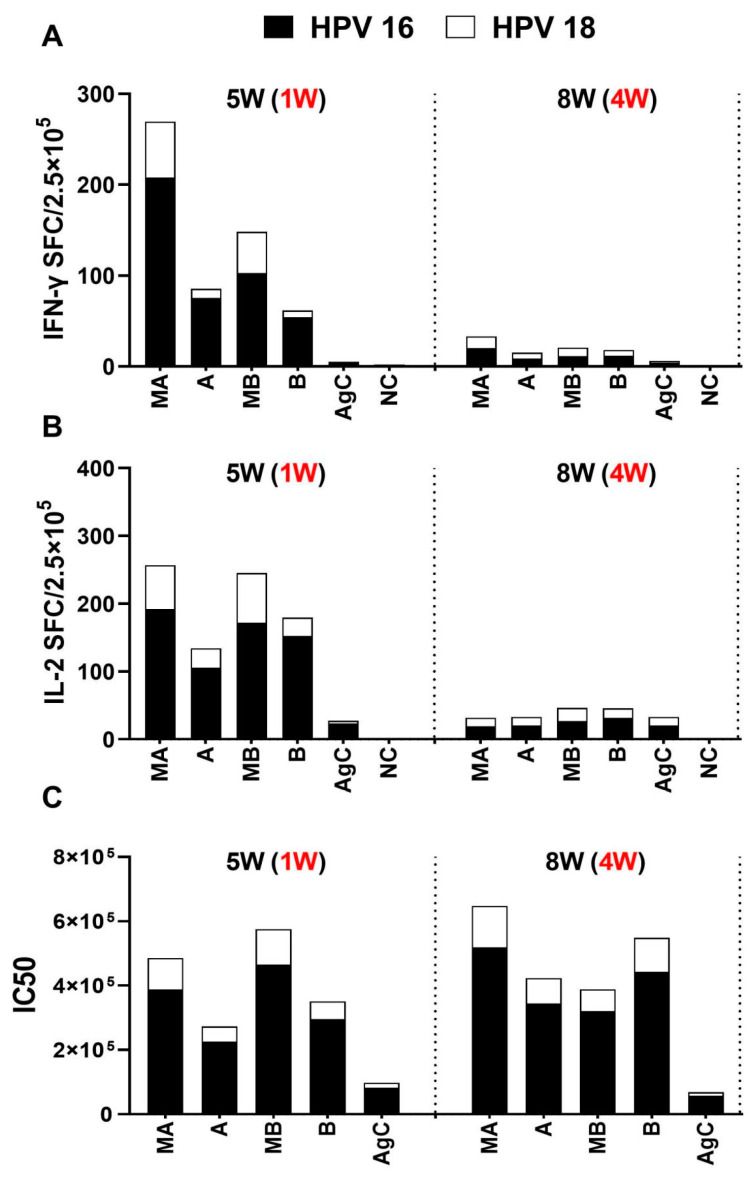
Three doses of different adjuvant combination vaccines induced an immune response to HPV types 16 and 18 L1 VLP antigens. The mice were immunized three times intramuscularly (i.m.) during a period of 2 weeks, either with HPV types 16 and 18 L1 VLPs alone or in combination with adjuvants. PBS was administered to mice in the negative control group. Spleen lymphocytes and sera were collected 1 and 4 weeks following the third immunization. (**A**): Both HPV16 and HPV18 L1 VLP-specific IFN-γ-producing splenocytes were evaluated using ELISpot assays. (**B**): Both HPV16 and HPV18 L1 VLP-specific IL-2-producing splenocytes were assessed by ELISpot assays. (**C**): Sera HPV16 and 18 antibody titers were detected using pseudovirus-based neutralization assays. The black font outside parentheses indicates the time from primary immunity to sacrifice, and the red font in parentheses indicates the time from boost immunity to sacrifice.

The in vivo experiments revealed that when two doses of low-dose aluminum hydroxide (formulation A) were combined with the BCG-CpG-DNA adjuvant, a Th1-immune response was induced. However, further improvement is needed to increase the impact on antibody production. To address this, a third homologous booster immunization was given to the mice. Following the booster immunization, HPV16- and 18-specific IFN-γ and IL-2 secretion increased in response to all BC02-adjuvanted vaccines. However, there was a slight downward trend observed 4 weeks after the last immunization (Figure 3A,B). Coadministration of the two adjuvants resulted in increased antibody titers. Significant increases were observed in mice that received the combination of low-dose aluminum hydroxide and BCG-CpG-DNA along with HPV16 and 18 L1 VLP antigens (MA group). These high antibody titers persisted until 4 weeks after the last immunization (Figure 3C). 

### 3.3. Medium-Dose BCG-CpG-DNA Enhanced the Adjuvant Effect of Al(OH)_3_ in the Immune Response to HPV16 and 18 L1 VLPs

**Figure 4:**
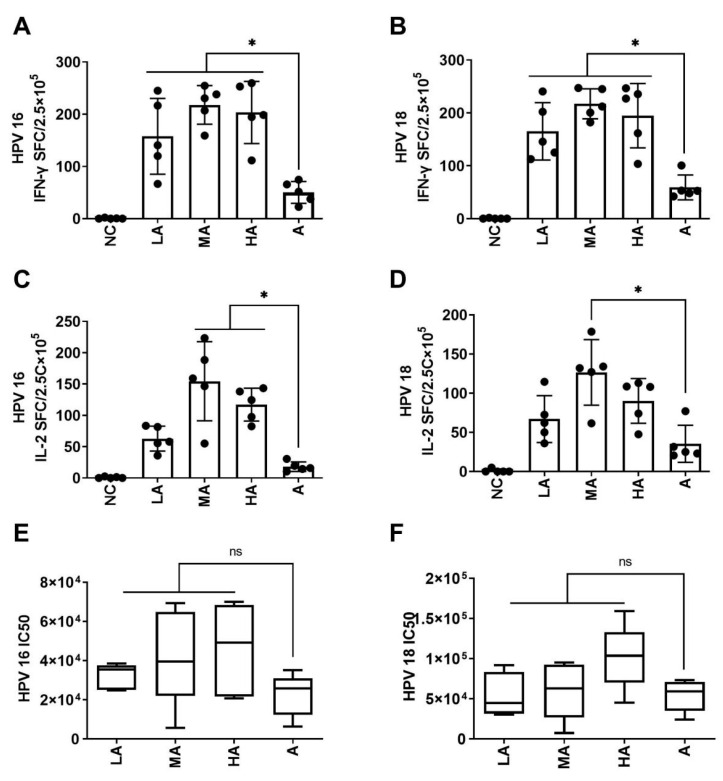
Comparison of the immunogenicity of HPV16 and 18 bivalent vaccines with different formulations of BC02 adjuvant. The mice were immunized two times intramuscularly (i.m.) during a period of 2 weeks, either with HPV types 16 and 18 L1 VLPs alone or in combination with adjuvants. PBS was administered to mice in the negative control group. Spleen lymphocytes and sera were collected 1 week following the second immunization. (**A**,**B**): Both HPV16 and HPV18 L1 VLP-specific IFN-γ-producing splenocytes were evaluated using ELISpot assays. (**C**,**D**): Both HPV16 and HPV18 L1 VLP-specific IL-2-producing splenocytes were assessed by ELISpot assays. (**E**,**F**): Sera HPV16 and 18 antibody titers were detected using pseudovirus-based neutralization assays. * indicate a significant difference between adjuvanted vaccines or between adjuvanted vaccines and unadjuvanted antigens, respectively. *p* < 0.05.

The above results demonstrated the success of combining low-dose aluminum hydroxide (25 µg, formulation A) with BCG-CpG-DNA as a novel formulation. This formulation was used to prepare HPV16 and 18 bivalent vaccines for further comparison of immune responses with different doses of BC02 compound adjuvant. The production of HPV16 and 18 L1 VLP-specific IFN-γ was dramatically higher in mice that received formulation A with low (LA), medium (MA), and high (HA) dose BCG-CpG-DNA adjuvant compared to those who received the Al(OH)_3_ adjuvant alone (LA vs. A _(16-IFN-γ)_: *p* = 0.027; MA vs. A _(16-IFN-γ)_: *p* = 0.001; HA vs. A _(16-IFN-γ)_: *p* = 0.002; LA vs. A _(18-IFN-γ)_: *p* = 0.005; MA vs. A _(18-IFN-γ)_: *p* < 0.0001; HA vs. A _(18-IFN-γ)_: *p* = 0.001, Figure 4A,B). Similarly, the production of HPV16 L1 VLP-specific IL-2 was higher in the MA and HA groups compared to the Al(OH)_3_ adjuvant alone group (MA vs. A _(16-IL-2)_: *p* = 0.0003; HA vs. A _(16-IL-2)_: *p* = 0.006, Figure 4C). However, the production of HPV18 L1 VLP-specific IL-2 showed a significant effect only for the MA group compared with the Al(OH)_3_ adjuvant alone group (MA vs. A _(18-IL-2)_: *p* = 0.003, Figure 4D).

When comparing anti-pseudoviral neutralizing antibodies, mice immunized with all BC02-adjuvanted vaccines exhibited increased antibody levels against HPV16 and 18, but these levels were similar to those in mice receiving Al(OH)_3_-adjuvanted vaccine (all *p* > 0.05, Figure 4E,F). 

### 3.4. Persistence of BC02-Adjuvanted Vaccine Immune Responses

**Figure 5:**
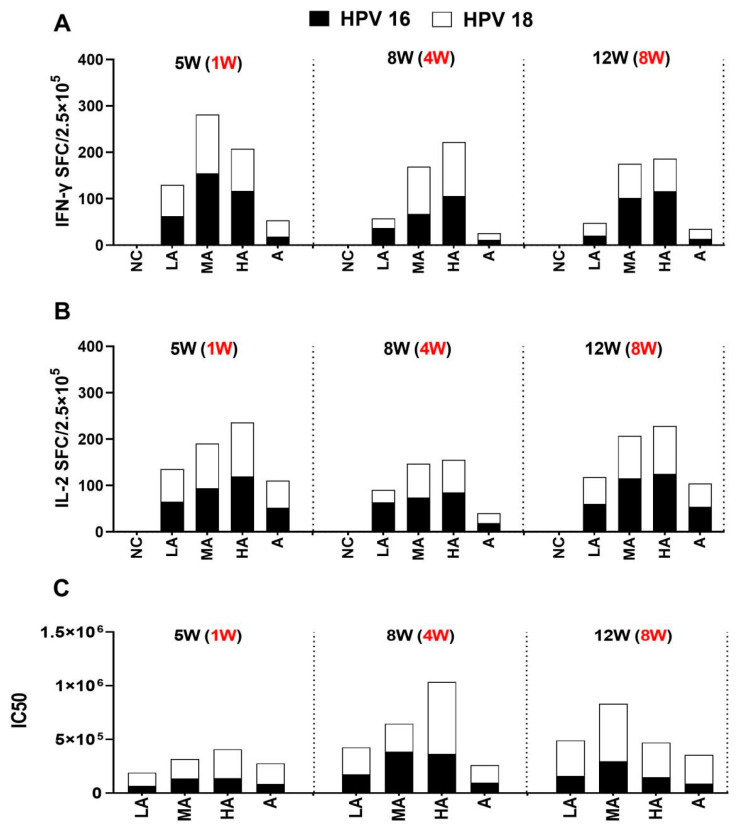
Three doses of distinct adjuvant combination vaccines induced immune responses to HPV16 and 18 L1 VLP antigens. The mice were immunized three times intramuscularly (i.m.) during a period of 2 weeks, either with HPV types 16 and 18 L1 VLPs alone or in combination with adjuvants. PBS was administered to mice in the negative control group. Spleen lymphocytes and sera were collected 1, 4 and 8 weeks following the third immunization. (**A**): Both HPV16 and HPV18 L1 VLP-specific IFN-γ-producing splenocytes were evaluated using ELISpot assays. (**B**): Both HPV16 and HPV18 L1 VLP-specific IL-2-producing splenocytes were assessed by ELISpot assays. (**C**): Sera HPV16 and 18 antibody titers were detected using pseudovirus-based neutralization assays. The black font outside parentheses indicates the time from primary immunity to sacrifice, and the red font in parentheses indicates the time from boost immunity to sacrifice.

According to the results mentioned above, the two doses of the experimental bivalent vaccine showed an improvement in the cellular immune response. However, the stimulation of antibodies still differed from that of the Al(OH)_3_-adjuvanted vaccine. Splenic IFN-γ and IL-2 secretion were measured at 1, 4, and 8 weeks after the final immunization and were found to be higher in all groups that received the BC02 compound adjuvant (LA, MA, and HA) compared to those who received the Al(OH)_3_ adjuvanted vaccine (Figure 5A,B). This indicates that the bivalent BC02-adjuvanted HPV vaccine induced a sustained preservation of Th1-type immunity over an extended period.

Additionally, the strength and persistence of antibody responses were also enhanced in the LA, MA, and HA groups compared to the two-dose immunizations. Titers of anti-HPV16 and 18 pseudovirus-neutralizing antibodies were maintained for 8 weeks after the last immunization in the MA group (Figure 5C).

## 4. Discussion

The presence of HPV DNA and the virus-transforming protein in almost all cervical cancer cells highlights the potential of HPV protein to elicit an immune response during vaccine immunotherapy. HPV vaccines can be categorized as prophylactic or therapeutic. Prophylactic vaccines target HPV capsid protein L1 or L1/L2 chimerism and are commonly used to induce humoral immunity in individuals who have not been previously exposed to HPV. Therapeutic vaccines, on the other hand, target the HPV primary oncogenic factors, E6 or E7, and aim to induce cellular immunity in patients with pre-existing infections and associated pre-invasive lesions. Both prophylactic and therapeutic vaccines consist of two essential components: target antigens and adjuvants.

HPV types 16 and 18 are recognized as the predominant HR HPVs responsible for cervical cancers and head and neck cancers. Globally, HPV16 accounts for 50% of HPV detections, while HPV18 accounts for 14% [2,16,17]. A meta-analysis showed a high-risk HPV infection rate of 19.0% among women in mainland China, with types 16, 52, 58, 53, and 18 exhibiting the highest infection rates [18,19]. In addition, HPV16 and HPV18 were the most prevalent subtypes across all age groups, and HPV16 has been associated with 46% of cervical intra-epithelial neoplasia 2/3 (CIN2/3) cases in China [20]. However, the prevalence of other HPV subtypes varied among age groups [21]. Therefore, HPV types 16 and 18 were chosen in this study. Reducing the cost of vaccine production and expanding the range of HPVs covered are both challenges to optimizing the prevention of HPV-associated malignancies. L1 capsomers, which remain stable at room temperature and do not require refrigeration, are an attractive option. Additionally, VLP vaccines can be administered through needle-free routes, such as transdermal application [22] and nasal inhalation [23]. The current commercially available HPV prophylactic vaccines are manufactured from L1 VLPs using eukaryotic expression systems, such as Saccharomyces cerevisiae cells or baculovirus-based Trichoplusia insect cells. However, in the present study, a more cost-effective alternative derived from *E. coli* was used as the source for the target antigen. Previous studies have shown that L1 capsids produced by the prokaryotic expression system can stimulate antibody production in animal models [24,25]. The bivalent VLP vaccine used in the current study, derived from *E. coli*, was successful in stimulating the production of anti-HPV 16 and anti-HPV 18 antibodies.

BCG-CpG-DNA, a novel biological adjuvant, possesses independent intellectual property rights and originates from the unmethylated CpG motif-containing DNA fragments from the Bacillus Calmette-Guerin genome. It demonstrates robust adjuvant properties in various species. Previous research has shown that *BCG-CpG-DNA* activates the innate immune response via TLR9 receptor-mediated activation of both NF-κB and MAPK pathways [26]. Additionally, it exhibits promising adjuvant performance in COVID-19 DNA vaccines [27]. BC02, a second-generation adjuvant, was generated by combining *BCG-CpG-DNA* with the inorganic salt, Al(OH)_3_. It stimulates both Th1 and Th2 immune responses to antigens derived from mycobacterium tuberculosis, toxoplasma gondii, or varicella-zoster virus [28,29,30,31,32,33]. The current work evaluated the potential of BC02 as an adjuvant for HPV 16 and 18 bivalent vaccines for the first time. It was found that the vaccine prepared with Al(OH)_3_ or BC02 compound adjuvant had elevated levels of IFN-γ and IL-2 compared to the naked antigen, but the BC02-based vaccine elicited a more robust Th1 cytokine response. These findings highlight the necessity of incorporating adjuvants in HPV 16 and 18 bivalent VLP vaccines and demonstrate that the cellular immunity response to BC02 vaccines is superior to that of Al(OH)_3_-adjuvanted vaccines. BCG-CpG-DNA was shown to be compatible with both low-dose (25 µg, formulation A) and high-dose (50 µg, formulation B) Al(OH)_3_, but the low-dose formulation had better performance in both 2- and 3-dose immunizations. This suggests that the binding of BCG-CpG-DNA to Al(OH)_3_ could reduce the amount of metal adjuvant, resulting in a stronger cellular immune response, particularly concerning HPV 16 antigen-specific IFN-γ levels, with improved safety. 

The study successfully optimized the compatible BCG-CpG-DNA dose in the compound adjuvant of the bivalent HPV 16 and 18 vaccines with formulation A by comparing it with the positive test control (PTC). The BC02-adjuvanted group induced higher HPV 16 and 18 antigen-specific IFN-γ and IL-2 levels compared to Al(OH)_3_ adjuvant or naked antigen in the two-shot immunization program. The optimal effect was achieved at 10 µg BCG-CpG-DNA, consistent with previous results. Although the anti-HPV16 and anti-HPV18 antibodies produced by the use of the BC02 adjuvant had lower titers than the PTC group in the two-dose immunization program, they showed persistent elevation after the addition of the third dose. This may be attributed to the mechanism by which Al(OH)_3_ adsorbs antigens, prolonging antigen release and action time. However, the BC02-adjuvanted bivalent HPV type 16 and 18 vaccines have not undergone additional validation in the mouse infection model, which represents a significant breakthrough for our future research.

## 5. Conclusions

The current study investigated the BC02 compound adjuvant in combination with the HPV VLP vaccine. The cellular and humoral immunities were examined at different time points, and the optimal formulation of the bivalent HPV 16 and 18 L1 VLP vaccine was determined. BC02 emerges as a promising adjuvant for the domestic production of an independent, innovative HPV vaccine.

## Figures and Tables

**Table 1:** Immunological grouping and dose of mice.

Dosage for the Preparation of Mouse Immunization	Group Codes
2 μg HPV16 + 2 μg HPV18 + 25 μg Al + 7.5 μg CpG	LA
2 μg HPV16 + 2 μg HPV18 + 25 μg Al + 10 μg CpG	MA
2 μg HPV16 + 2 μg HPV18 + 25 μg Al + 12.5 μg CpG	HA
2 μg HPV16 + 2 μg HPV18 + 25 μg Al	A
2 μg HPV16 + 2 μg HPV18 + 50 μg Al + 10 μg CpG	MB
2 μg HPV16 + 2 μg HPV18 + 50 μg Al	B
2 μg HPV16 + 2 μg HPV18	AgC
PBS	NC

Al: Al(OH)_3_, CpG: BCG-CpG-DNA, LA: Low-dose CpG-formulation A, MA: Medium-dose CpG-formulation A, HA: High-dose CpG-formulation A, A: Formulation A, MB: Medium-dose CpG-formulation B, B: Formulation B, AgC: Antigen control, NC: Negative control.

## Data Availability

Not applicable.
